# Comfort level of caregivers of cancer patients receiving palliative care

**DOI:** 10.1590/1518-8345.2521.3029

**Published:** 2018-08-09

**Authors:** Maisa Vitória Gayoso, Marla Andréia Garcia de Avila, Thays Antunes da Silva, Rúbia Aguiar Alencar

**Affiliations:** 1 Master’s student, Faculdade de Medicina de Botucatu, Universidade Estadual Paulista “Júlio de Mesquita Filho”, Botucatu, SP, Brazil. Scholarship holder at Fundação de Amparo à Pesquisa do Estado de São Paulo (FAPESP), Brazil.; 2 PhD, Professor, Faculdade de Medicina de Botucatu, Universidade Estadual Paulista “Júlio de Mesquita Filho”, Botucatu, SP, Brazil.; 3 Physician, Serviço de Terapia Antálgica e Cuidados Paliativos, Hospital das Clínicas, Botucatu, SP, Brazil.

**Keywords:** Palliative Care, Caregivers, Family, Neoplasms, Scales, Patient Care Team

## Abstract

**Objective:**

To verify the association between the level of comfort of the caregiver and socio-demographic variables related to caregiving, and the patient’s functional status and symptoms.

**Method:**

Cross-sectional study with non-probabilistic intentional sample. The instruments Palliative Performance Scale (score 0 to 100%), Edmonton Symptom Assessment Scale (symptom scores from zero to ten) and Holistic Comfort Questionnaire (total score ranging from 49 to 294 and mean score from 1 to 6) were used. The relationship between comfort scores and independent variables was calculated by multiple linear regression.

**Results:**

Fifty informal caregivers participated in the study - 80% were female, 32% were 60 years old or older, 36% were children of the patient, 58% had paid work and 60% did not have help in the care. The mean overall comfort was 4.52 points. A better functional status of the patients was associated with higher levels of comfort of the caregivers. Older caregivers who received helped in the care activities presented higher comfort scores.

**Conclusion:**

The level of comfort of caregivers of cancer patients receiving palliative care was associated with socio-demographic variables and patients’ functional status and symptoms.

## Introduction

Human aging leads to an increase in chronic-degenerative diseases, such as cancer. In 2017, 1,688,780 new cancer cases and 600,920 cancer deaths are projected to occur in the United States, with an incidence rate 20% higher in men than in women^1^. According to the literature, by 2025 there will be 25 million annual cases of cancer in the world, with highest increases expected in low income countries^2^.

Along with these transitions, the changes in therapy and the technological developments achieved in the second half of the last century have led to an increase in the longevity of patients with incurable diseases. This transition reveals new needs for health care, which leads to a new way of thinking about patient care^3^.

According to the World Health Organization (WHO), in 2011, 20.4 million people needed palliative care in the world, of which 69% were 60 years old or older^4^. This new epidemiological profile stimulates discussions about the process of dying with dignity. Considering this principle, the practice of “Palliative Care”, as defined by WHO in 1990 and updated in 2002, is expressed as^4^ “[...]an approach that improves the quality of life of patients and their families facing the problem associated with life-threatening illness, through the prevention and relief of suffering by means of early identification, impeccable assessment and treatment of pain and other problems, physical, psychosocial and spiritual”.

Palliative Care aims to treat death as a natural and expected process associated with the disease. It addresses not only the patients, but also their relatives, who witness the patient’s transition from a healthy person to one with limitations and who can also become ill^4^ ^-^ ^5^. Family members are often the main caregivers, who experience situations of pain, anxiety and the potential loss of a loved one. This group is essential to support the patient, but it is also impacted by the disease, and their suffering must also be addressed and relieved^6^. 

The family caregiver, also known as informal caregiver, is someone who does not get paid to take on this role and who provides non-professional care, that is, without technical training. The literature shows the importance of improving the care offered to these caregivers, since they may be overburdened with their role^7^ ^-^ ^8^.

It is important to create strategies to evaluate the impact that the interaction with the disease can have on the caregiver, to conduct research that can improve the care offered to this relative and to develop means to support the nucleus of care. The use of holistic tools can improve the care provided to the caregiver in clinical practice and emphasize that the multidimensional aspects related to caregivers’ comfort have received little attention in the literature^9^ ^-^ ^11^.

The concept “comfort” still does not have a consensual definition in the literature, but it can be defined as a subjective state of well-being occurring at any time during the health/disease process. According to a Theory of Comfort created in 1991^12^, comfort comprises four contexts of human needs: physical comfort refer to bodily sensations (such as pain); psycho-spiritual comfort relates to internal self-awareness (such as faith, self-esteem and sexuality); socio-cultural comfort relates to interpersonal relationships (such as relationship with family, financial issues and relationship with the health team); and environmental comfort relates to external surroundings (such as lighting, odor and temperature)^12^ ^-^ ^13^.

The WHO establishes that continuous care must be extended to family members. However, according to the literature, studies addressing caregivers are recent, with more of 70% of them published after 2008^14^. Currently, there is scarce literature on the comfort of family caregivers of adult cancer patients receiving palliative care. Thus, this study aims to verify the association between the comfort level of caregivers of cancer patients receiving palliative care and socio-demographic variables, variables related to the care performed, and the patient’s functional status and symptoms. 

## Method

This is a cross-sectional study with non-probabilistic intentional sample. It was conducted from June to August, 2016, in the outpatient clinic and home care of the Palliative Care team of a tertiary Hospital in a city in the state of São Paulo, Brazil. This period comprehended the curricular internship of one of the researchers in the Multi-professional Residency Program in Adult and Older Adult Health in the Botucatu Medical School, State University of São Paulo “Julio de Mesquita Filho”. 

Informal caregivers of cancer patients receiving palliative care were included in the research, when they did not get paid for this function, they were 18 years old or older and were identified as primary caregiver by the patient and/or their relatives. Caregivers of inpatients or patients who were in their first appointment with the Palliative Care service were excluded from the study. During this period, there were approximately 120 cancer patients who were followed-up in the palliative care service. Thus, the sample consisted of 50 caregivers and included all of those who fulfilled the inclusion criteria in the period of the curricular internship of one of the researchers. 

The data were initially collected through a questionnaire with socio-demographic characteristics. Afterwards, the Palliative Performance Scale (PPS), Edmonton Symptom Assessment Scale (ESAS) and the Holistic Comfort Questionnaire - caregiver (HCQ-caregiver) were used.

The PPS is an instrument developed in 1996 by the Victoria Hospice, Canada, with the objective of assessing the functional status of the patient and understanding the evolution of the disease. The scale has eleven levels, from zero to 100%, divided in intervals of 10. A 100% level means that the patient is fully functional, and zero means death^15^.

ESAS is an important tool for symptom assessment developed in Edmonton, Canada in 1991 and translated and adapted for use in Brazil in 2013. It is a small questionnaire with nine specific symptoms, divided in physical symptoms (pain, tiredness, nausea, drowsiness, lack of appetite, shortness of breath), and psychological symptoms (depression, anxiety and well-being). Each symptom is rated from 0 to 10, zero meaning that the patient is at the best state possible and the symptom is absent and 10 that it is of the worst possible severity^16^.

The HCQ-caregiver was created in 2001^17^ and validated in Brazil in 2015^18^. It is an instrument that evaluates the comfort of these professionals unidimensionally. This evaluation serves as basis for planning better interventions in the care provided to the caregiver. Higher total scores indicate a higher level of comfort. If you add up the scores assigned to each sentence of the questionnaire, the total score of the instrument ranges from 49 to 294 points. Many studies use the average score that ranges from one to six points, which is the total score divided by the 49 questions. The HCQ-caregiver comprises four dimensions of comfort: the physical, the psycho-spiritual, the socio-cultural and the environmental. The maximum score that can be obtained in the physical comfort dimension is 42, in the psycho-spiritual dimension it is 90, in the socio-cultural dimension it is 96 and in the environmental dimension it is 66. Some questions were constructed as negative sentences. For those to be statistically evaluated and considered in the total score of the instrument, it is necessary to reverse the result at the time of tabulation^17^ ^-^ ^19^. 

The questionnaires can be self-administered or applied by interviewer. In this study, by preference of the participants, the questionnaires were applied by the researcher

The caregivers were invited to participate in the study while awaiting the consultation of patients with the Palliative Care team or at the time of home care. It should be noted that the interview was always conducted in a quiet environment, in a reserved room in the institution or in the interviewee’s house, in a private place with only the participant and the researcher. Each interview lasted an average of 40 minutes and all participants signed the informed consent form. 

The statistical power was calculated using simple random sampling, type I error= 0.05, estimation of standard deviations of the PPS, outcomes and estimates of linear regression coefficients of the fitted models. A test power of over 80% was estimated for general comfort and for the other dimensions of comfort.

The Statistical Package for the Social Sciences (SPSS) V21.0 was used to analyze the data. The relationship between the comfort scores and the independent variables was analyzed by multiple linear regression. All effects and relationships associated with values ​​of p<0.05 were considered significant. The project was approved by the Research Ethics Committee of the institution through opinion number 1,576,496 (CAAE protocol no. 55366216.0.0000.5411). 

## Results

In the sample of 50 informal caregivers most were female (80%). The care of children (36%) and participation of caregivers aged 60 years or over (32%) were highlighted. The caregiver’s ages were from 18 to 80 years, with a median age of 52.5 years.

All caregivers reported they had a religion and lived with someone. Regarding the professional situation, 29 (58%) had paid work (not as caregiver). Regarding level of education, a median of eight years of study was found. The time since diagnosis, as well as the duration of care provided to cancer patients receiving palliative care, was from one to 240 months, with a median of 24 months. Of the total, 60% reported they did not receive assistance to perform the care.

All patients in the study had cancer. The most prevalent type of cancer was breast cancer, with nine cases (18%), followed by prostate and bowel cancer, with five cases each (10%), and uterus, lung and esophagus cancer, with three cases each (6%).

The PPS assessment showed that caregivers classified the functional status of the patients as 50 to 70% in 25 cases (50%), followed by 80 to 100% in 14 cases (28%) and 0 to 40% in 11 cases (22%). The maximum value was 100% and the minimum was 20%, with a median of 60%. 

Regarding the ESAS, as scored by the caregiver, the patients had the following median scores: 6.5 for fatigue and lack of appetite; 06 for pain, anxiety and drowsiness; 05 for depression and malaise; 0.5 for nausea; and zero for shortness of breath.

The HCQ-caregiver presented a maximum score of 275 points and a minimum of 136 points, with a median of 230.5 points. The mean overall comfort of the caregiver, in this study, was 4.52 points: physical comfort obtained 4.78 points; psycho-spiritual comfort obtained 4.56 points; sociocultural comfort obtained 3.85 points; and environmental comfort obtained 5.28 points. 

It was evidenced that the higher the age of the caregiver, the greater their overall comfort score, which had a significant association (p=0.018). Each additional year in the age of the caregiver increases overall comfort by 1.35 points. In addition, help to deliver care had a significant relationship with comfort (p= 0.004). The overall comfort score is 43 points higher for those who receive help when compared to caregivers who do not have any assistance, according to Table 1.

**Table 1:** Linear regression adjusted for the caregiver’s holistic comfort. Botucatu, SP, Brazil, 2016

Variable	B^*^	95%CI^†^		P^‡^
Caregiver’s age	1.35	0.24	2.46	0.018*§*
Gender	7.62	-31.56	46.79	0.695
Years of education	2.63	-1.30	6.56	0.182
Paid work	-7.44	-39.65	24.78	0.641
Time since diagnosis	0.16	-0.51	0.83	0.635
Length of time assisting patient	-0.22	-0.84	0.41	0.490
Receives help in the care	43.69	14.70	72.69	0.004*§*
PPS^||^	1.23	0.33	2.13	0.009*§*
Pain	0.76	-5.67	7.19	0.811
Tiredness	-5.41	-11.23	0.41	0.067
Nausea	-1.16	-6.55	4.23	0.664
Depression	-1.13	-5.75	3.48	0.620
Anxiety	1.99	-2.65	6.64	0.388
Drowsiness	3.22	-0.69	7.13	0.103
Lack of appetite	1.96	-3.53	7.45	0.473
Shortness of breath	2.85	-1.86	7.56	0.227
Malaise	1.66	-4.05	7.36	0.558

*B- Regression coefficient; †CI- Confidence interval; ‡P- Probability of significance; *§*p< 0.05; ||PPS- Palliative Performance Scale

A significant association was observed between the PPS scores and the HCQ-caregiver (p=0.009). The greater the functional status of the patient, the higher the degree of comfort of the caregiver. An extra point in the PPS increases the overall comfort by an average of 1.23 points, according to Table 1.

The analysis of the domains of the HCG-caregiver separately demonstrated that only the PPS had a significant association with physical comfort (p=0.006). The higher the PPS score, the greater the physical comfort of the caregiver. Thus, an additional point on the PPS scale increases physical comfort by an average of 0.23 points, as shown in Table 2.

**Table 2:** Linear regression adjusted for the caregiver’s physical comfort. Botucatu, SP, Brazil, 2016

Variável	B*	95%CI^†^		P^‡^
Caregiver’s age	0.174	-0.023	0.372	0.081
Gender	1,945	-5.039	8.928	0.575
Years of education	0.051	-0.65	0.751	0.884
Paid work	0.04	-5.703	5.783	0.989
Time since diagnosis	-0.001	-0.121	0.118	0.981
Length of time assisting patient	-0.003	-0.115	0.109	0.954
Receives help in the care	4.793	-0.375	9.961	0.068
PPS^||^	0.233	0.073	0.394	0.006^||^
Pain	0.068	-1.079	1.214	0.905
Tiredness	-0.477	-1.514	0.561	0.357
Nausea	-0.454	-1.414	0.506	0.343
Depression	-0.114	-0.936	0.708	0.779
Anxiety	0.227	-0.601	1.054	0.581
Drowsiness	0.3	-0.397	0.998	0.387
Lack of appetite	0.215	-0.764	1.193	0.658
Shortness of breath	0.358	-0.482	1.197	0.392
Malaise	0.451	-0.566	1.468	0.373

*B- Regression coefficient; ^†^CI- Confidence interval; ^‡^P- Probability of significance; ^||^PPS- Palliative Performance Scale; ^||^p< 0.05

The psycho-spiritual dimension has a significant association with the caregiver’s age (p=0.012). The greater the age of the caregiver, the greater their degree of comfort and each additional year in the age of the caregiver increases their psycho-spiritual comfort by an average of 0.54 points. Receiving help for the care also demonstrates significant influence when associated with the psycho-spiritual dimension (p=0.019), since the psycho-spiritual comfort score is 13.23 points higher for those who receive help, as it can be seen in Table 3.

**Table 3:** Linear regression adjusted for the caregiver’s psycho-spiritual comfort. Botucatu, SP, Brazil, 2016

Variable	B*	95%CI^†^		P^‡^
Caregiver’s age	0.545	0.13	0.96	0.012^*§*^
Gender	-2.315	-17.019	12.39	0.751
Years of education	0.961	-0.514	2.437	0.194
Paid work	-4.006	-16.098	8.085	0.505
Time since diagnosis	0.054	-0.197	0.305	0.664
Length of time assisting patient	-0.142	-0.378	0.094	0.229
Receives help in the care	13.234	2.352	24.116	0.019^*§*^
PPS^||^	0.461	0.122	0.799	0.009^*§*^
Pain	-0.83	-3.245	1.584	0.489
Tiredness	-2.577	-4.762	-0.392	0.022*§*
Nausea	-0.228	-2.249	1.794	0.820
Depression	0.48	-1.251	2.211	0.576
Anxiety	0.572	-1.171	2.314	0509
Drowsiness	1.046	-0.423	2.514	0.157
Lack of appetite	0.748	-1.312	2.808	0.465
Shortness of breath	1.277	-0.491	3.045	0.151
Malaise	0.744	-1.397	2.885	0.484

*B- Regression coefficient; †CI- Confidence interval; ‡P- Probability of significance; *§*p< 0.05; ||PPS- Palliative Performance Scale

The PPS score was also significantly associated with this dimension (p=0.009): an extra point on the PPS scale increases the caregiver’s psycho-spiritual comfort by an average of 0.46 points. Patient tiredness measured through the ESAS had a significant influence on psycho-spiritual comfort (p=0.022), with a negative result for the caregiver’s comfort. An additional point in the patient’s tiredness score decreases by 2.57 points the caregiver’s psycho-spiritual comfort, according to Table 3.

Socio-cultural comfort was influenced by the help received to deliver care, with a significant association (p=0.005). The socio-cultural comfort score is on average 12.72 points higher for those who receive help from third parties than for those who do not receive help. Another variable that had significant influence on this dimension was the PPS score (p=0.012). An additional point in the PPS score increases the socio-cultural comfort of the caregiver by an average of 0.35 points, according to Table 4.

**Table 4:** Linear regression adjusted for the caregiver’s sociocultural comfort. Botucatu, SP, Brasil, 2016

Variable	B*	95%CI^†^		P^‡^
Caregiver’s age	0.227	-0.101	0.555	0.168
Gender	5.595	-6.021	17.211	0.334
Years of education	0.841	-0.325	2.006	0.152
Paid work	-0.331	-9.883	9.222	0.944
Time since diagnosis	0.064	-0.134	0.263	0.515
Length of time assisting patient	-0.033	-0.22	0.153	0.719
Receives help in the care	12.722	4.126	21.319	0.005^*§*^
PPS^||^	0.352	0.084	0.619	0.012^*§*^
Pain	1.031	-0.876	2.939	0.279
Tiredness	-1.457	-3.183	0.269	0.095
Nausea	-0.474	-2.071	1.123	0.549
Depression	-1.114	-2.482	0.253	0.107
Anxiety	0.658	-0.719	2.034	0.338
Drowsiness	1.075	-0.085	2.235	0.068
Lack of appetite	0.747	-0.88	2.375	0.357
Shortness of breath	0.754	-0.643	2.151	0.280
Malaise	0.029	-1.662	1.721	0.972

*B- Regression coefficient; †CI- Confidence interval; ‡P- Probability of significance; §p< 0.05; ||PPS- Palliative Performance Scale

The environmental comfort is significantly influenced by age (p=0.003). The older the caregiver, the greater their environmental comfort, and each additional year in the age of the caregiver increases the environmental comfort by an average of 0.40 points. The help received also had a significant association with this dimension (p=0.000). The environmental comfort score is on average 12.94 points higher for those who receive help, as it can be observed in Table 5.

**Table 5:** Linear regression adjusted for the caregiver’s environmental comfort. Botucatu, SP, Brazil, 2016

Variable	B*	CI95%^†^		P^‡^
Caregiver’s age	0.403	0.144	0.662	0.003^*§*^
Gender	2.391	-6.774	11.555	0599
Years of education	0.778	-0.142	1.697	0.095
Paid work	-3.138	-10.674	4.398	0.403
Time since diagnosis	0.041	-0.116	0.197	0.599
Length of time assisting patient	-0.037	-0.184	0.11	0.613
Receives help in the care	12.944	6.162	19.727	0.000^*§*^
PPS^||^	0.181	-0.03	0.392	0.090
Pain	0.492	-1.013	1.997	0.510
Tiredness	-0.901	-2.263	0.461	0.187
Nausea	-0.004	-1.264	1.256	0.995
Depression	-0.386	-1.465	0.693	0.471
Anxiety	0.537	-0.549	1.623	0.321
Drowsiness	0.799	-0.116	1.715	0.085
Lack of appetite	0.247	-1.037	1.531	0.698
Shortness of breath	0.462	-0.64	1.564	0.400
Malaise	0.434	-0.9	1.769	0.512

*B- Regression coefficient; †IC- Confidence interval; ‡P- Probability of significance; §p< 0.05; ||PPS- Palliative Performance Scale

## Discussion

In this research, as it can be found in the literature, the caregivers are predominantly female (80%) and in the age group of 60 years or more, which shows that care is still very frequently delegated to adult women^9^ ^,^ ^17^ ^,^ ^20^ ^-^ ^21^.

Regarding the degree of kinship, as found in other studies, children are the main responsible for care, followed by spouses, stressing that care still remains within the nuclear family^20^. In a study conducted in Campinas in 2010 with 133 caregivers, 43.1% of them were the children of the patients^19^.

According to other authors, religion is an important factor for maintaining the quality of life of the caregiver and the patient^22^ ^-^ ^23^. It serves as support during moments of crisis, and it is important for coping, adaptation and well-being. In this study, all participants reported having a religion; however, this aspect was not significantly associated with the comfort of the caregiver. Faith is a way of finding strength to face the disease, even with the impossibility of healing^24^.

Regarding the professional situation, as found in other studies, most caregivers have a paid work, so care represents “second shift” situation. This reduces the caregiver’s rest time, as in a study in Canada, which found that 42.9% of caregivers also had a paid work^20^.

The time since the diagnosis and the time since the caregiver initiated care are similar in this research, which was also found in a study carried out in São Paulo^9^. A study carried out in Portugal considers duration of caregiving as a positive factor for the caregiver, believing that it generates mechanisms to cope and adapt to care activities, even though these variables did not demonstrate any significant statistical association with the caregiver comfort in this research^25^.

The most prevalent type of cancer in the study was the breast cancer, which, according to a 2015 study, is the most commonly found in women in Brazil and in the world, and the second most common in the general population^26^. Prostate cancer is the second most prevalent type of cancer in men and the fourth most common type in the world, after bowel cancer, which is the third most common in the world^2^. A study conducted in Germany in 2016 also shows breast, prostate and bowel cancers as the most prevalent^5^.

Among the symptoms presented by the patients according to the caregiver, pain and tiredness were the most common. However, statistically, the patients’ symptoms did not influence the overall comfort level of the caregiver. Only tiredness had a negative association with the psycho-spiritual dimension. It was not possible to compare these data with other studies because of the lack of studies relating the patient’s symptoms to the comfort or quality of life of the caregiver.

Regarding the level of comfort of the caregiver, a mean score of 4.52 points was found, a result similar to a study carried out in São Paulo in 2014. This can be associated with the characteristics of the service, which is specialized in palliative care and provides, in addition to hospital care, outpatient and home care activities. However, this value is still far from reaching the desired score of 6 in the HCQ-caregiver^9^.

Nursing includes an ongoing process of assessing the needs of individuals. Literature considers comfort as one of the centers of this intentional evaluation, which leads nursing professional to constantly attempt to satisfy the basic needs of the individual and promote comfort. The fact that comfort is an individual experience related to the way each subject experiences and interprets situations, providing maximum comfort to individuals is a challenge difficult to achieve^12^ ^,^ ^27^. And this is true not only for caregivers of cancer patient. According to a study carried out in Lisbon with caregivers of patients with various chronic diseases, the mean general comfort score was 4.23, not reaching the maximum of the questionnaire^13^.

It was possible to perceive that receiving help in this care is a positive factor for overall comfort, because when the task is shared, the burden becomes lighter and the caregiver can have more time for self-care^11^. In literature, social support is considered an important factor for the quality of life of caregivers, influencing their emotional and physical health^28^. In the study, most caregivers do not have any help for this function (60%), a result different from a study conducted in Porto Alegre in 2015^20^.

The nurse is an important actor in the construction of care plans aimed at promoting well-being and treating the informal caregiver not only as an ally in care, but as an individual that must receive care. The literature proposes actions that can support the dynamics of informal care, such as rotation of family members, training of the informal caregiver for practical care activities, and ongoing encouragement for the self-care of these caregivers^29^ ^-^ ^30^.

Another relevant variable is the caregiver’s age. Older caregivers presented a higher level of comfort when compared to younger caregivers. This may be because their life experience increases their coping capacity^9^. Another possibility is that more experienced caregivers can provide a resilient care, a fact that might be a new aspect to be studied. A 2014 study shows the resilience of the older adult as a result of the inconveniences and difficulties experienced during the trajectory of their lives, making them more fit to deal with critical moments^31^. 

The functional status of the patient was one of the variables most associated with the comfort level of the caregiver, meaning that the patient’s level of independence is related higher levels of comfort of the caregiver, which was also found in other studies^9^ ^,^ ^32^. The need for a greater number of care activities is a heavy burden on the caregivers, generating negative repercussions in the physical and psychological dimensions^33^.

The separate analysis of the dimensions of comfort showed socio-cultural comfort as the one with the lowest levels, with 3.85 points. This leads to reflection on the need to provide better support to the caregiver. Researches reveal the need for a multi-professional team to prepare the caregiver and clarify the doubts related to the patient’s illness. This can be a way to reduce the caregiver’s anxiety and increase the quality of the care provided^9^ ^,^ ^34^. A clear communication with the family is fundamental, given that uncertainty regarding the disease process is one of the factors that most impairs the well-being of the caregiver^35^.

Finally, it is necessary to reduce the distance between the health team and the caregiver, who needs to be technically and emotionally prepared to perform their role with comfort and consequently to improve the quality of the care provided^7^.

The results presented highlight the relevance of studies of this nature and the importance of perceiving the caregiver as an important actor in the care of the patient. The HCQ-caregiver presented good internal consistency and allowed identifying factors that influence caregiver comfort.

It should be noted that the non-probabilistic sample is a limitation of the study. Finally, further studies on the comfort of caregivers and their quality of life are necessary. 

## Conclusion

The mean score of the comfort level of caregivers of cancer patients receiving palliative care was close to the maximum score of the instrument, which represents a good comfort level for the caregiver. In addition, the overall comfort of the caregiver was associated with socio-demographic variables and with the patient’s functional status and symptoms. 

A better functional status of the patients was associated with a higher level of comfort of the caregivers. In addition, older caregivers who can rely on other people’s help to provide care have higher comfort scores. On the other hand, tiredness presented by the cancer patient negatively influences the psycho-spiritual comfort. 

The socio-cultural dimension presented the lowest scores, emphasizing the need for a closer contact between the caregiver and the health team, who should provide clarification about the patient’s illness. The family caregiver should be prepared for this care and supported during the difficulties encountered in the caring process.

Comprehensive care for cancer patients receiving palliative care must also include assistance to their caregivers, since it is evident that the caregiver’s comfort can be influenced positively or negatively by several factors. 

This study contributes to the advancement of knowledge in the field of palliative care, since it, in a relevant way, brings focus to the care offered to informal caregivers of cancer patients receiving palliative care and provides resources for further research in this area.
